# GYY4137 Attenuates Sodium Deoxycholate-Induced Intestinal Barrier Injury Both In Vitro and In Vivo

**DOI:** 10.1155/2019/5752323

**Published:** 2019-10-13

**Authors:** Zeyang Chen, Jianqiang Tang, Pengyuan Wang, Jing Zhu, Yucun Liu

**Affiliations:** Division of General Surgery, Peking University First Hospital, Peking University, 8 Xi Shiu Street, Beijing 100034, China

## Abstract

**Objectives:**

Substantial studies have demonstrated that an elevated concentration of deoxycholic acid (DCA) in the colonic lumen may play a critical role in the pathogenesis of intestinal barrier dysfunction and inflammatory bowel disease (IBD). The purpose of this study was to investigate the protective effects of GYY4137, as a novel and synthetic H_2_S donor, on the injury of intestinal barrier induced by sodium deoxycholate (SDC) both in vivo and in vitro.

**Methods:**

In this study, Caco-2 monolayers and mouse models with high SDC concentration in the lumen were used to study the effect of GYY4137 on intestinal barrier dysfunction induced by SDC and its underlying mechanisms.

**Results:**

In Caco-2 monolayers, a short period of addition of SDC increased the permeability of monolayers obviously, changed distribution of tight junctions (TJs), and improved the phosphorylation level of myosin light chain kinase (MLCK) and myosin light chain (MLC). However, pretreatment with GYY4137 markedly ameliorated the SDC-induced barrier dysfunction. Being injected with GYY4137 could enable mice to resist the SDC-induced injury of the intestinal barrier. Besides, GYY4137 promoted the recovery of the body weight and intestinal barrier histological score of mice with the gavage of SDC. GYY4137 also attenuated the decreased expression level of TJs in mice treated with SDC.

**Conclusion:**

Taken together, this research suggests that GYY4137 preserves the intestinal barrier from SDC-induced injury via suppressing the activation of P-MLCK-P-MLC2 signaling pathway and increasing the expression level of tight junctions.

## 1. Introduction

The fact that between 1/4 and 1/3 of the Americans can be classified as obese is closely associated with the intake of high-fat diet [1]. Recently, it was shown that high-fat diet impairs intestinal barrier and has correlation with the occurrence of inflammatory bowel disease (IBD) [1–3]. High-fat feeding can increase the concentration and proportion of deoxycholic acid (DCA) in feces, that is the main ingredient of hydrophobic secondary bile acids and whose cytotoxic and intestinal barrier disrupting effects have been fully reported [2–6]. One of the main determinants of intestinal permeability is the intercellular tight junctions (TJs), preventing the translocation of antigens through the epithelium [7]. These antigens play significant roles in the pathogenesis of severe clinical outcomes (e.g., IBD and endotoxemia). Previous studies have reported that DCA or sodium deoxycholate (SDC) could induce altered expression and localization of TJs, leading to the intestinal barrier injury, as well as increased incidence of IBD [8, 9].

In recent years, hydrogen sulfide (H_2_S), which is traditionally believed to be a toxic gas, has been shown to be a signal molecule, exhibiting a variety of physiological functions which are beneficial to the body [10–12]. Recent st udies reported the potential gastrointestinal protective and ulcer-healing properties of H_2_S [12, 13]. In addition, a previous study showed GYY4137 as a H_2_S donor, that can release low dose of H_2_S for a long time and that preserves the intestinal barrier function by significantly inhibiting the decreased expression and altered localization of TJs in the context of endotoxemia [14]. However, the effect of GYY4137 on SDC-induced intestinal barrier injury has not been fully elucidated, and the underlying mechanisms have still remained known.

The chronic intake of high-fat feeding can increase the luminal concentration of SDC, which can lead to the increased intestinal permeability. In our study, protective effects of GYY4137 on the function of the intestinal barrier in the context of short period and relatively long-term exposure of SDC at the level of cell and animal were elaborated. Our results may exert new insights on potential therapeutic approaches for SDC-related intestinal barrier dysfunction.

## 2. Materials and Methods

### 2.1. Chemicals and Regents

In our research, SDC, GYY4137, FITC-dextran (4 KDa, FD-4), and FITC-dextran (40 KDa, FD-40) were purchased from Sigma-Aldrich (St. Louis, MO, USA). Primary antibodies for immunofluorescence and western blotting were purchased from companies as follows: Occludin (Thermo Fisher Scientific, Waltham, MA, USA); MLCK (Abcam, Cambridge, UK); P-MLCK (Abcam, Cambridge, UK); ZO-1 (Cell Signaling Technology, Danvers, MA, USA); MLC2 (Cell Signaling Technology, Danvers, MA, USA); and P-MLC2 (Cell Signaling Technology, Danvers, MA, USA).

### 2.2. Cell Culture

Experiments were conducted with Caco-2 cells which were purchased from American Type Culture Collection (ATCC, Manassas, VA, USA) between passages 28 and 34. Cells were grown at 37°C and cultured in Dulbecco's Modified Eagle's Medium (DMEM) supplemented with 4.5 mg/mL glucose, 10% fetal bovine serum (FBS), 25 mmol/L HEPES (4-(2-hydroxyethyl)-1-piperazineethanesulfonic acid), 50 U/mL penicillin, and 50 U/mL streptomycin as previously described [14–16]. In order to grow on the transwell, 105 cells with high density were seeded on filters with 0.4 *μ*m pore size (Corning Inc., Corning, NY, USA). In the basolateral compartments of transwells, the medium containing various concentrations of GYY4137 was added with or without 2 mM SDC in the apical compartments.

### 2.3. Transepithelial Electrical Resistance (TEER) Measurements

The TEER was measured as described previously [17]. An epithelial volt-ohm meter (ERS-2; Merck Millipore, Burlington, MA, USA) was used to measure the changes of TEER of Caco-2 monolayers. About 21 days after confluence when the epithelial resistance of monolayers reached to 350–550 Ω·cm^2^ [18], different reagents were added to the transwell as indicated. The TEER was assessed until approximate values were recorded consecutively in three times.

### 2.4. Paracellular Marker FITC-Dextran 40 kDa (FD-40) Flux Measurements

Paracellular permeability was measured using a previous method [19, 20]. After the treatment described beforehand, monolayers were washed with phosphate-buffered saline (PBS) solution, and then 1 mg/mL FD-4 solution diluted by Hank's balanced salt solution was added to the apical compartments for 2 h. After taking 100 *μ*L solution from the basolateral compartments, in 492 nm excitation and 520 nm emission filter, the fluorescence of FD-40 flux was assessed with Synergy H2 microplate reader (BioTek Instruments Inc., Winooski, VT, USA). Besides, calibration curves were drafted by serial dilution of FD-40 to determine FD-40 concentrations.

### 2.5. Western Blot Analysis

A previously reported method was used for the total protein extraction of Caco-2 monolayers [21]. In vivo, the total protein of the mucosa (2 cm) in the proximal colon was extracted using a method described previously with some modifications [14, 22]. The concentration of proteins was measured using bicinchoninic acid (BCA) method (Thermo Fisher Scientific, Waltham, MA, USA). Subsequently, the electrophoresis of extracts involving equivalent quantities of proteins (25 *μ*g) was conducted in 6% or 10% polyacrylamide gel, and then various proteins were transferred to polyvinylidene difluoride (PVDF) membranes. At room temperature, the membrane was blocked for nonspecific binding for 1 h (5% bovine serum albumin (BSA) in TBS-Tween 20 buffer) and then incubated overnight at 4°C with primary antibodies (1 : 1000 dilution). Subsequently, with the corresponding secondary antibodies (1 : 1000 dilution), the membrane was incubated at room temperature for 1 h. Finally, blots were developed with electrochemiluminescence (ECL) detection reagents (Merck Millipore, Burlington, MA, USA) and visualized by Syngene GeneGenius gel imaging system (Syngene, Frederick, MD).

### 2.6. Immunofluorescence of ZO-1 and Occludin in Caco-2 Monolayers

After being treated as indicated previously, cellular distribution of ZO-1 and Occludin was visualized by immunofluorescence as previously reported [14, 21]. In brief, after being rinsed with PBS, filters were fixed in 100% methanol overnight at −20°C and 100% acetone at −20°C for 1 min. Subsequently, at room temperature, filters were blocked with 1% BSA for 2 h and then treated with anti-mouse Occludin (4 *μ*g/ml) and anti-rabbit ZO-1 (6 *μ*g/ml) at 4°C overnight. After being washing with PBS, filters were incubated with goat anti-mouse IgG conjugated to Alexa555 (Molecular Probes, Eugene, OR, USA) and goat anti-rabbit IgG conjugated to Alexa488 (Molecular Probes, Eugene, OR, USA) in 1% BSA at room temperature for 1 h and then rinsed with PBS. In the next step, the Prolong Gold Antifade Reagent (Molecular Probes, Eugene, OR, USA) was used, and cells were stored at 4°C in dark until analysis. Under Fluoview 1000 confocal microscope (Olympus, Tokyo, Japan), the fluorescence was visualized.

### 2.7. Animals

After being purchased from Vital River Inc. (Beijing, China), male C57BL/6 mice (8-weeks old) were raised in the containment unit with access to water and food *ad libitum* at the Laboratory Animal Center at the Peking University First Hospital (Beijing, China). Before any treatment, the mice had a week to adapt to the environment. The mouse model of SDC with high concentration in the enteric cavity was established by gastric perfusion of SDC (250 mg/kg body weight) to mice once daily for 5 consecutive days, which maintained from the 1^st^ day to the 5^th^ day. In addition, thirty-six mice were divided into four groups randomly as follows: control; GYY4137 alone; SDC; and SDC + GYY4137. Mice in the control group were injected intraperitoneally with PBS and gavaged with sterile water. Mice in the GYY4137 group were injected intraperitoneally with 50 mg/(kg·d) GYY4137 for 5 consecutive days. Mice in the SDC group were given by gavage of 250 mg/(kg·d) SDC for consecutive 5 days. Mice in the SDC + GYY4137 group were given by gavage of 250 mg/(kg·d) SDC and injected intraperitoneally with 50 mg/(kg·d) GYY4137 for 5 consecutive days. Body weight was daily recorded until the 5^th^ day. All mice were euthanized 12 h after final administration. All procedures of this study were approved by the Institutional Review Board of Peking University First Hospital.

### 2.8. Measurement of Intestinal Permeability in Mice

The epithelial barrier permeability of mice was evaluated by assessing the concentration of FD-4 in plasma as previously described [23]. Briefly, 12 h after final administration, mice were gavaged with FD-4 (60 mg/100g body weight). Blood was collected 4 h after FD-4 gavage by cardiac puncture in EDTA-coated tubes. Subsequently, the plasma was collected by centrifuging at 3000 rpm for 15 min at 4°C, and its fluorescent signal of plasma was assessed with a Synergy H2 microplate reader (BioTek Instruments Inc., Winooski, VT, USA) using 492 nm excitation and 520 nm emission filters. Standard curves were generated by serial dilution of FD-40 to determine FD-40 concentrations.

### 2.9. Histological Assessment

The proximal colons (3 per each group) collected from mice in different groups were excised and embedded in paraffin. Sections (thickness, 4 *μ*m) were cut and stained with hematoxylin and eosin (H&E). Images were observed using a Zeiss Image light microscope (magnification: 20x; Carl Zeiss AG, Oberkochen, Germany). The degree of histopathologic changes was graded as previously described: Score 0, normal histological findings. Score 1, mucosa: villus blunting, loss of crypt architecture, sparse inflammatory cell infiltration, vacuolization, and edema; muscle layer: normal. Score 2, mucosa: villus blunting with fattened and vacuolated cells, crypt necrosis, intense inflammatory cell infiltration, vacuolization, and edema; muscle layer: normal. Score 3, mucosa: villus blunting with fattened and vacuolated cells, crypt necrosis, intense inflammatory cell infiltration, vacuolization, and edema [24].

### 2.10. Statistical Analysis

The results were expressed as mean ± standard error of the mean (SEM) and analyzed using a Student's *t*-test for unpaired data and one-way analysis of variance (ANOVA) to compare groups whenever required by GraphPad Prism 5.0 software. A *P* value < 0.05 was considered to be statistically significant.

## 3. Results

### 3.1. The Effects of SDC on the TEER of Caco-2 Monolayers

**Figure 1:**
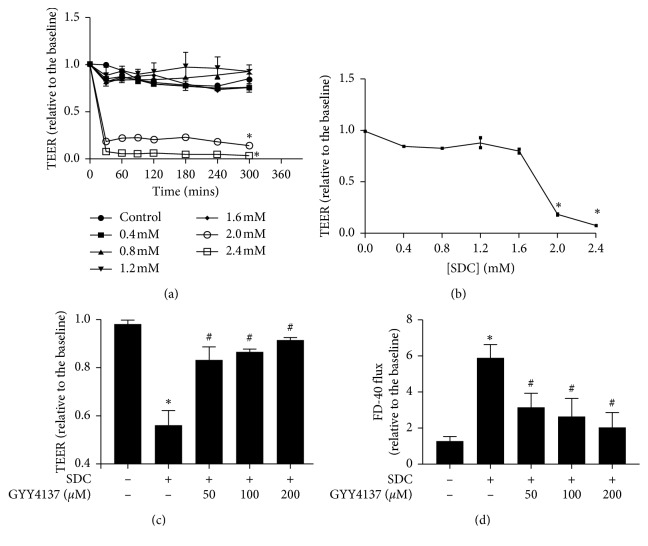
Destructive effects of SDC on Caco-2 monolayer barrier function and GYY4137 ameliorates intestinal epithelial barrier dysfunction induced by SDC. (a) Caco-2 monolayers were treated with different concentrations (0–2.4 mM) of SDC. 2 Mm or greater concentration could significantly decrease the TEER at 30 min. (b) Dose-response of the TEER of Caco-2 monolayers treated with different concentrations of SDC for 30 min. Concentration ≥2 mM significantly degraded the TEER. (c) Caco-2 monolayers were preincubated with or without 50, 100, and 200 *μ*M GYY4137 for 48 h and then treated in the presence or absence of 2 mM SDC for 30 min. GYY4137 significantly attenuated TEER reduction induced by SDC treatment. (d) Caco-2 monolayers were treated as described. The increase of FD-40 flux induced by SDC was significantly attenuated by GYY4137 treatment. Results were expressed as mean ± SEM (*n* = 3). ^*∗*^*P* < 0.05 vs. control.

We assessed the barrier disruption by a reduction in the TEER after the application of SDC in the Caco-2 monolayer. We tested different concentrations (0–2.4 mM) of SDC and found that 2 mM concentration or greater was sufficient to significantly decrease the TEER (*P* < 0.05, Figure 1(a)). In a relatively short time, 30 min, the TEER declined rapidly to under 50% of its initial level. At the end of 30 min, the downtown of TEER induced by SDC was pronounced enough in minimum dose. The TEER did not show further remarkable drop over time (Figure 1(a)).

We want to find a minimum concentration of SDC, which could cause a >50% fall in the TEER of Caco-2 monolayer at 30 min. After adding various concentrations of SDC to the apical compartments for 30 min, the concentration of 2 mM would be used in the subsequent experiments (Figure 1(b)).

### 3.2. The Protective Effects of GYY4137 on SDC-Induced Injuries of Intestinal Barrier

TEER and FD-40 flux were used to assess the monolayer barrier function. After being exposed to 2 mM SDC for 30 min, TEER decreased (*P* < 0.05, Figure 1(c)), while FD-40 flux significantly increased (*P* < 0.05, Figure 1(d)). In order to explore the protective effects of GYY4137 on SDC-induced injuries of barrier function, the monolayers were pretreated with increasing doses of GYY4137 (50, 100, and 200 µM) for 48 h followed by being exposed to 2 mM SDC for 30 min. Preincubation with different concentrations of GYY4137 could in a dose-dependent manner notably alleviate the fall of TEER and the increase of FD-40 flux induced by SDC (Figures 1(c) and 1(d)). GYY4137, at 200 *μ*M concentration, produced the most significant effect, and thereafter, 200 *μ*M GYY4137 was used in the following experiments.

### 3.3. The Effects of GYY4137 and SDC on Tight Junctions

**Figure 2:**
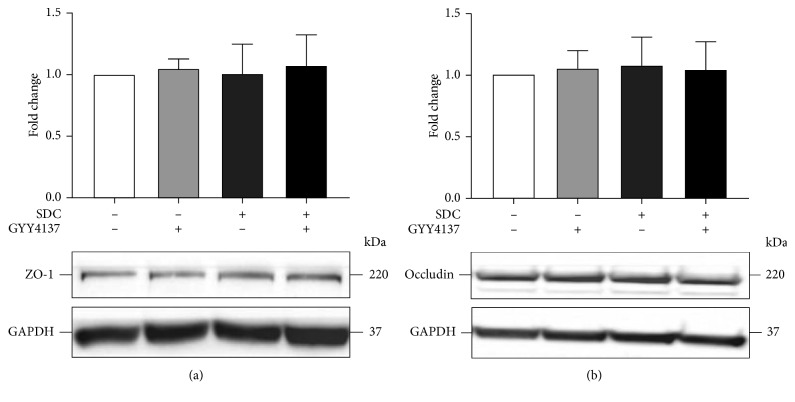
The effects of GYY4137 and SDC on the expression level of TJs in monolayers. Caco-2 monolayers were preincubated with or without 200 *μ*M GYY4137 for 48 h and then treated in the presence or absence of 2 mM SDC for 30 min. The total protein of monolayers was harvested after treatment for western blot assay. Results were expressed as mean ± SEM (*n* = 3).

The decrease of expression level of TJs and altered localization of TJs can both cause injury to the intestinal epithelial barrier function [14]. However, GYY4137 or exposure of SDC for 30 min could not exert significant influence on the expression level of TJs (Figure 2). Thus, immunofluorescences of ZO-1 and Occludin were assessed to examine whether SDC could change the permeability of monolayer by altering the distribution of TJs.

**Figure 3:**
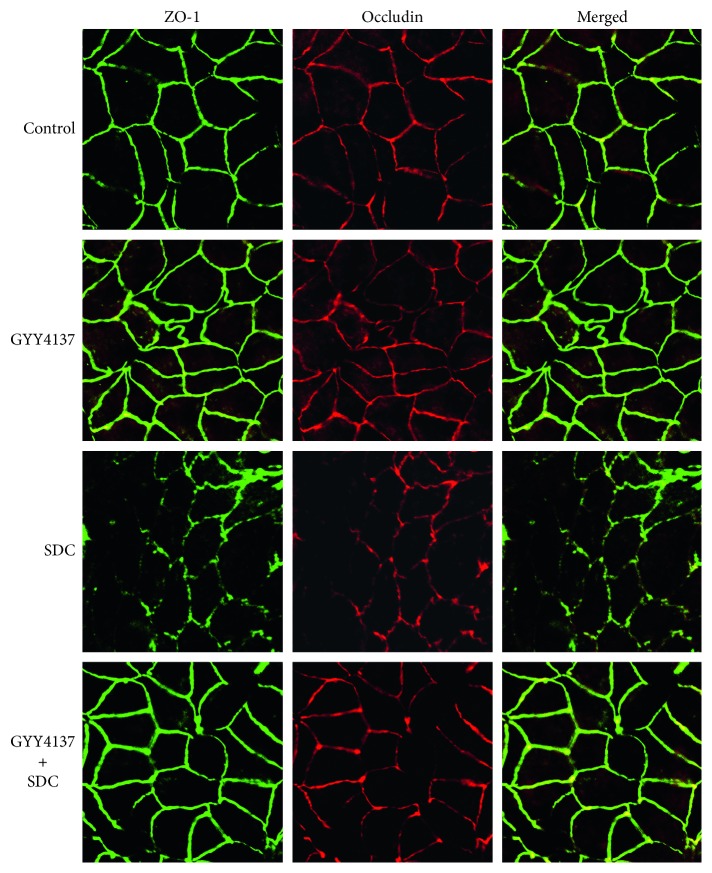
The effects of GYY4137 and SDC on the localization of TJs in monolayers. ZO-1 and Occludin were stained by immunofluorescence. The altered localization of TJs induced by SDC was ameliorated by GYY4137.

In general, smooth edges and typical “chicken wire” were normal shapes of immunofluorescences of ZO-1 and Occludin [14]. However, after being added with 2 mM SDC for 30 min, the cellular edges became serrated. Network patterns of ZO-1 and Occludin were disrupted with areas of discontinuous and irregular labeling intensity. These results indicated that SDC caused abnormal localization of the TJs. However, pretreatment with GYY4137 attenuated the changes caused by SDC exposure. With preincubation of 200 *μ*M GYY4137 for 48 h, ZO-1 and Occludin remained in a “chicken wire” shape after adding SDC to the Caco-2 monolayer (Figure 3).

### 3.4. The Effects of GYY4137 on SDC-Induced Activation of P-MLCK-P-MLC2 Signaling Pathway

**Figure 4:**
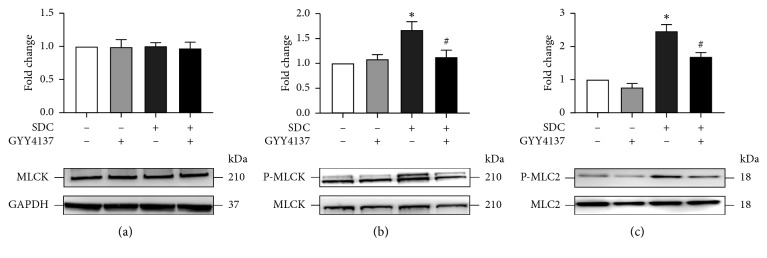
The effects of GYY4137 on the status of P-MLCK-P-MLC2 signaling pathway in Caco-2 monolayers with short-term SDC treatment. Caco-2 monolayers were treated as described in Figure 3. (a–c) GYY4137 inhibited SDC-induced increased phosphorylation of MLCK and MLC2. Results were expressed as mean ± SEM (*n* = 3). ^*∗*^*P* < 0.05 vs. control. ^#^*P* < 0.05 vs. SDC.

Previous studies have reported the pivotal role of MLCK-P-MLC2 pathway in the physiological and pathological regulation of the localization of TJs [14, 25]. However, GYY4137 or treatment of SDC for 30 min, that is a relatively short time, had no significant influence on the expression level of MLCK (Figure 4(a)). On the other hand, addition of SDC transiently increased MLCK phosphorylation (Figure 4(b)), as the active form of MLCK and a key regulator of tight junction distribution which could increase the phosphorylation level of MLC2 [26, 27].

After the treatment of Caco-2 monolayers with SDC for 30 min, there was an obvious increase in the phosphorylation level of MLCK and MLC2 compared with control (*P* < 0.05). However, compared with Caco-2 monolayers treated with SDC, pretreatment with GYY4137 for 48h significantly inhibited the increase of phosphorylation level of MLCK and MLC2 (*P* < 0.05, Figures 4(b) and 4(c)). This result showed that GYY4137 suppressed P-MLCK-P-MLC2 signaling pathway and then ameliorated SDC-induced intestinal barrier destruction.

### 3.5. GYY4137 Ameliorates SDC-Induced Weight Loss of Mice

**Figure 5:**
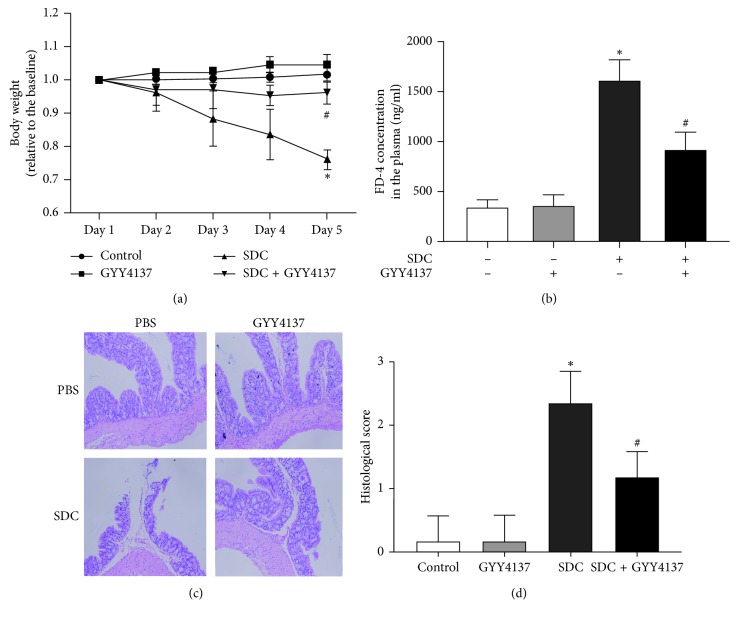
The effects of GYY4137 on the body weight, barrier function, and histological score of mice with the gavage of SDC. (a) GYY4137 obviously inhibited the decreased body weight of mice treated with SDC. (b) GYY4137 remarkably ameliorated the broken intestinal barrier featured by increased FD-4 flux in mice treated with SDC. (c) GYY4137 attenuated the mucosal damage caused by SDC. (d) GYY4137 significantly attenuated the increased histological score in mice with the gavage of SDC. All experiments were performed using 6 mice per experimental group and repeated at least three times. Results were expressed as mean ± SEM (*n* = 6). ^*∗*^*P* < 0.05 vs. control. ^#^*P* < 0.05 vs. SDC.

Body weight sharply decreased due to SDC treatment, in contrast to control and GYY4137 groups (Figure 5(a)). The weight loss due to SDC treatment was significantly improved in mice administrated with GYY4137 on the 5th day (*P* < 0.05, Figure 5(a)).

### 3.6. GYY4137 Had Protective Effects on the Intestinal Barrier Function in Mice Treated with SDC

In mice treated with SDC, the FD-4 concentration in the plasma was significantly higher than that in the control (*P* < 0.05, Figure 5(b)). However, the concentration of FD-4 was markedly decreased by cotreatment of GYY4137 (*P* < 0.05, Figure 5(b)). These results suggested that GYY4137 attenuated the injuries induced by SDC on intestinal barrier function.

### 3.7. GYY4137 Improved the Histological Status in Mice Treated with SDC

Cotreatment with GYY4137 attenuated the histological damage of colon epithelium in mice with SDC treatment, featured by villus stunting, deciduous epithelial cells, crypt disruption, and discrete submucosal (Figures 5(c) and 5(d)).

### 3.8. GYY4137 Inhibited SDC-Induced Decreased Expression of TJs in Mice

**Figure 6:**
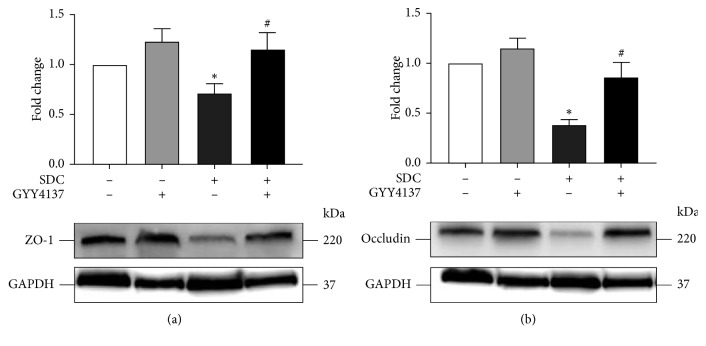
The effects of GYY4137 on the expression level of TJs of mice with the gavage of SDC. GYY4137 significantly inhibited the decreased expression of ZO-1 and Occludin in the proximal colon of mice with the gavage of SDC. All experiments were performed using 6 mice per experimental group and repeated at least three times. Results were expressed as mean ± SEM (*n* = 6). ^*∗*^*P* < 0.05 vs. control. ^#^*P* < 0.05 vs. SDC.

The treatment by SDC for a relatively short time, 30 min, could not affect the expression level of TJs. However, in mice, exposure to SDC for a relatively long time, 5 days, significantly decreased the expression level of TJs. In the proximal colon, GYY4137 notably ameliorated the reduced expression level of TJs (*P* < 0.05, Figure 6).

## 4. Discussion

TJs, whose expression level and localization can determine intestinal barrier function, are an essential component of intestinal mucosal mechanical barrier [7, 14]. A large body of evidence suggests that intestinal barrier dysfunction plays a pivotal role in the pathogenesis of multiple enteropathies (e.g., IBD). People with long-term high-fat feeding have higher rates of IBD [1–3] and intraluminal concentrations of hydrophobic secondary bile acids [2, 3]. The high concentration of DCA, the main secondary bile acid compromising approximately 20% of bile acids [28, 29], can damage the function of intestinal barrier via altering expression [30] and localization [28, 31] of TJs. Thus, it is a prospective research to seek potential therapeutic reagents for individuals with high concentrations of bile acids in the lumen who may have long-term high-fat diet.

In this study, we used Caco-2 monolayers and the effects of SDC on the intestinal barrier function were investigated. Previous studies have reported that SDC can break the barrier function of monolayers in a relatively short period, in about 1 h [28, 32], in which our results were consistent with previous studies. Our research indicated that 2 mM concentration of SDC or greater was sufficient to decrease the TEER to <50% of its initial level at 30 min. Previous studies indicated that SDC can damage the epithelial cells by direct destructive effects and inducing apoptosis [33, 34]. To ensure the highest activity of cells, we selected a relatively short processing time and a low concentration, 30 min and 2 mM, in the subsequent experiments. Previous research has shown that bile acids exist in the human intestine at a concentration with a typical range from 2–6 mM [28]; therefore, doses of SDC used in this study fall within the physiologic range. Our study suggested that expose of Caco-2 monolayers to 2 mM SDC for 30 min did not affect the expression level of TJs. The immunofluorescence suggested that the principal factor in SDC-induced destruction of intestinal barrier at 30 min was the altered localization of TJs.

GYY4137, as a novel and ideal H2S donor, is stable in vitro and in vivo. Compared with NaHS, in longer term, GYY4137 can release H_2_S stably in the physiological context [14, 35]. H_2_S, as a new type of gaseous signal molecule, has anti-inflammatory and antiapoptosis influences on multiple tissues [36, 37]. However, the influences of GYY4137 on the SDC-induced injury of barrier function and the underlying mechanisms need to be elucidated. A previous research revealed that GYY4137 could ameliorate intestinal barrier injury by regulating the expression level and localization of TJs in the context of endotoxemia [14]. In the present study, by in vitro utilizing Caco-2 monolayers, we realized that the pretreatment with GYY4137 for 48 h significantly attenuated the SDC-induced decrease of TEER and increase of FD-40 flux. GYY4137 also was able to ameliorate the altered localization of TJs caused by SDC.

Additionally, MLCK-P-MLC2 is a very classic signaling pathway to regulate barrier function by altering the distribution of TJs. Actin-myosin filaments are able to contract when the phosphorylation level of MLC2 increase, resulting in rapidly altered distribution of TJs and broken intestinal barrier [22, 25]. In our study, the findings of immunoblotting revealed that SDC notably upregulated the phosphorylation of MLC2. However, 30 min, SDC was unable to regulate expression level of MLCK. MLCK phosphorylation, a key regulator of the localization of TJs, representing the active form of MLCK, can increase phosphorylation of MLC2 [26, 27]. Moreover, SDC can enhance the phosphorylation level of MLCK, manifesting the involvement of P-MLCK-P-MLC2 pathway in SDC-caused barrier dysfunction in Caco-2 monolayers. Subsequently, we assessed the effects of GYY4137 on SDC-induced activation of P-MLCK-P-MLC2 signaling pathway. The findings showed that pretreatment with GYY4137 remarkably suppressed the activation of P-MLCK-P-MLC2 signaling pathway induced by SDC.

In order to further verify our results, we established a mouse model with high SDC concentration in the lumen. It was disclosed that GYY4137 could significantly suppress the sharply decreased body weight, as one of the representative symptoms of broken intestinal barrier function, which was induced by SDC treatment. The permeability of intestinal barrier was also examined by assessing the plasma concentration of FD-4. The results showed that GYY4137 was able to protect the mice from the injuries of intestinal barrier caused by SDC. Histological assessment of the colon epithelium suggested that GYY4137 could promote the recovery of abnormal histopathological features induced by SDC. In addition, GYY4137 alleviated the decreased expression level of TJs caused by SDC.

In a relatively short time in vitro, SDC was able to break barrier function by regulating the localization of TJs. However, in a relatively long time in vivo, SDC exerted destructive effect on the intestinal barrier by decreasing the expression level of TJs. Furthermore, GYY4137 can affect both localization and expression level of TJs to exert protective effects on the intestinal barrier.

In summary, the present study illustrated that GYY4137 could ameliorate SDC-induced injuries of intestinal barrier both in vitro and in vivo, and the suppression of SDC-induced activation of P-MLCK-P-MLC2 signaling pathway and decreased expression level of TJs might be one of the underlying mechanisms of protective effects. Our research may provide a reliable basis for the application of GYY4137 against bile acids-related gut leakiness, and this study has also a great potential to become a novel therapeutic approach to prevent and remedy high-fat diet-related IBD.

## Data Availability

All data generated or analysed during this study are available from the corresponding author on reasonable request.
